# Evolutionary psychological perspectives on filicide are applicable in modern-day Norway

**DOI:** 10.3389/fpsyg.2023.1019020

**Published:** 2023-01-24

**Authors:** Vibeke Ottesen

**Affiliations:** ^1^Department of Psychosocial Science, Faculty of Psychology, University of Bergen, Bergen, Hordaland, Norway; ^2^Centre for Ecological and Evolutionary Synthesis, Faculty of Mathematics and Natural Sciences, University of Oslo, Oslo, Norway

**Keywords:** filicide, filicide-suicide, perpetrator psychopathology, discriminative parental investment, evolutionary psychology, universal validity, premises for testing validity

## Abstract

Current evolutionary psychological (EP) perspectives on filicide perpetration propose that it is an extreme behavioral manifestation of psychological mechanisms that evolved due to their function toward enabling ancestrally adaptive discriminative parental investment. Predictions concerning the characteristics traits of filicide derived from this hypothesis have been empirically supported cross-culturally. Still, it remains a theoretical and empirical question whether EP perspectives on filicide are applicable in societies where the general population is alleviated from ancestrally salient cues to reproductive conflict between individuals and children in their care and the opportunities for lethal caretaker behaviors are highly constricted. Compiling a national sample of filicide cases in modern-day Norway, the present study catered for testing whether EP perspectives on filicide apply in such a society. As predicted, the majority of incidents (79.5%) were associated with perpetrator psychopathology (psychotic episode or filicide-suicide). This further catered for testing the EP hypothesis that filicides associated with perpetrator psychopathology will be characterized by traits that contradict an ancestrally adaptive logic for discriminant parental investment. A full series of predictions derived from this hypothesis was supported as filicides with this association included no step-parental perpetrators and, when compared against filicides that were not associated with perpetrator psychopathology, had older victims and perpetrators and more often multiple victims. The findings confirm the potential applicability of EP perspectives on filicide in progressive and highly developed welfare states, thus lending support to their universal validity.

## 1. Introduction

In their pioneering theoretical and empirical work in the field of homicide research, evolutionary psychologists Daly and Wilson (1988b) explored whether reproductive conflicts (i.e., when the fitness interest of one individual conflicts with the fitness interest of another individual) might lie at the core of a range of homicide categories, including filicide. Referring to established knowledge in the field of evolutionary biology, Daly and Wilson (1980, 1988a,b) noted that caretaker-child-dyads hold the potential for reproductive conflicts with regard to the level of parental investment that is made. As parental resources were scarce in our species’ ancestral past, evolutionary selection processes would inevitably favor psychological mechanisms that motivate the individual to solve such conflicts by adjusting the level of investment made in a given child in accordance with the perceived prospects of successfully fostering the child to reproductive maturity over mechanisms that would motivate for non-discriminant parental investment. Further, Daly and Wilson argued that filicide may result when psychological mechanisms enabling discriminant parental investment are over-activated, thus causing the caretaker to lower investment to lethal levels. This perspective holds that filicide perpetration as an epiphenomenon of evolved psychological mechanisms selected for ancestrally adaptive, non-lethal discriminant parental investment (i.e., filicide perpetration is a by-product and not an adaptation). Offering a different perspective, evolutionary psychologists Buss (2005) and Duntley and Buss (2008, 2011) have argued that evolutionary selection processes would have favored psychological mechanisms that specifically motivated for filicide perpetration in our ancestral past over mechanisms that merely result in non-lethal adjustments in parental investment, as specifically lethal mechanisms would have protected the caretaker from potentially wasting resources more abruptly and effectively. From the shared hypothesis that filicide perpetration is underpinned by evolved psychological mechanisms enabling the individual to perform discriminant parental investment in the context of reproductive conflicts, the proponents of the two perspectives have argued that the characteristic traits of filicide will follow an ancestrally adaptive logic for discriminant parental investment.

A fundamental premise for proposing that a given psychological mechanism is an evolved adaptation, is that the mechanisms should be universally shared among humans and therefore found cross-culturally. If the EP hypothesis that filicide perpetration is underpinned by evolved psychological mechanisms enabling ancestrally adaptive discriminative parental investment is valid, then we should find this reflected in the characteristics of filicides cross-culturally. The predictions concerning the characteristics of filicide derived from the presented EP hypothesis have been empirically supported in a range of hunter-gatherer societies using data from ethnographic records (Daly and Wilson, 1988a,b). The predictions have further been empirically supported in relatively modernized societies. These societies include Canada, for the time periods 1974–1983 (Daly and Wilson, 1988a,b), 1974–1990 (Daly and Wilson, 1994; Wilson et al., 1995), and 1996–2003 (Harris et al., 2007), the United States, for the time period 1976–1994 (Weekes-Shackelford and Shackelford, 2004), England and Wales, for the time period 1977–1990 (Daly and Wilson, 1994; Wilson et al., 1995; Nobes et al., 2019), Italy, for the time period 1976–2010 (Camperio Ciani and Fontanesi, 2012), and Sweden, for the time period 1975–1995 (Daly and Wilson, 2001). Further, EP predictions have been empirically supported when tested in sub-samples of filicide, including a sample of filicides that occurred in Detroit (United States) in the year 1972 (Daly and Wilson, 1988a,b), samples of filicide-suicides that occurred in Chicago (United States) in the time periods 1870–1930 (Shackelford et al., 2008) and 1965–1994 (Shackelford et al., 2005), and a sample of filicides perpetrated by female patients hospitalized at the Mid-Hudson Forensic Psychiatric Hospital (United States) in the time period 1978–2000 (Stone et al., 2005). This cross-cultural empirical support for the EP informed predictions concerning the characteristics of filicide implies a support for the theoretical validity of the hypothesis they were derived from.

Having established the usefulness of EP perspectives in guiding the identification of significant characteristics of filicide in the above list of societies does not imply that the scholarly field of filicide research is eternally done with evaluating the universal validity of EP perspectives and that the potential contribution of such perspectives may make toward our scholarly understanding of filicide is conclusively fulfilled. For instance, after testing a selection of EP predictions in Swedish national samples of filicide, focusing mainly on the potential overrepresentation of stepfathers among filicide perpetrators, Temrin et al. (2000, 2004, 2011) and Nordlund and Temrin (2007) questioned the validity of the EP the prediction that as step-relationships are more vulnerable to reproductive conflicts than genetic relations they are also more vulnerable to the most extreme behavioral manifestations of discriminant parental investment, such as fatal abuse. However, as Daly and Wilson (2001) argued, it may be that the selected prediction merely does not apply in modern-day Sweden, rather than that EP perspectives on filicide are not theoretically valid. When testing the validity of the EP hypothesis concerning filicide perpetration, it is imperative to pay explicit attention to two premises concerning evolved psychological mechanisms that are as crucial as the premise of universality; namely that *the activation* of such mechanisms is contingent on ancestrally salient cues and that *the specific behavioral manifestations* of the mechanisms are contingent on what options individuals have and perceive in their given environment (e.g., Tooby and Cosmides, 1989).

So far, no study has paid explicit attention to these two latter premises when testing the validity of EP perspectives on filicide, as the focus of previous studies has solely been on exploring the premise of universality. The present study is an example of how such an informed test of the validity of EP perspectives on filicide perpetration can be done, as the ambition is to explore the applicability of such perspectives in guiding the identification of the characteristic traits of filicide in modern-day Norway. This society is a highly progressive and well-developed welfare state, where social, legal, and political structures alleviate the general population from ancestrally salient cues that might (over)activate evolved psychological mechanisms for discriminant parental investment, thus preventing potentially lethal behavioral manifestations of such mechanisms. In the present paper, I argue that there are numerous EP informed predictions concerning the characteristics of filicides that may nevertheless be applicable to this society. If these predictions are empirically supported in the present study, or indeed if they are not, this will contribute toward the evaluation of the universal validity of EP perspectives on filicide and thus to our scholarly understanding of the parental psychology that underpins filicide perpetration and how this psychology may be activated and manifest depending on a societies’ social, political, and legal structures.

## 2. Identifying EP predictions concerning the characteristic of filicide that apply to modern-day Norway

There are two variables that have been predicted and confirmed from EP perspectives to be strongly associated with the risk for filicide perpetration that might not be as relevant toward this risk in modern-day Norway; namely the age of a child and the household composition. In the following, I present the EP reasoning for why these two variables have been predicted to increase the risk for filicide, and why they nevertheless will not be as relevant in modern-day Norway. I will then argue that it follows from an EP perspective that in the vacuum of ancestrally salient cues to reproductive conflicts between individuals and children in their care in the general population and limited opportunities for lethal manifestations of such conflicts, that the majority of filicides in this society will occur in association with perpetrator psychopathology. I will then present a series of four EP predictions concerning the characteristics of such filicides. The present study will test whether these combined five predictions apply to modern-day Norway.

### 2.1. Age of the child

Within the field of evolutionary biology, there are three conditions argued to bias caretakers toward conditional discrimination against their youngest offspring, thus favoring their older offspring, with regard to parental investment (Trivers, 1972, 1974; Alexander, 1979). First, the youngest offspring are dependent on the greatest levels of parental investment for survival and healthy growth. Second, the reproductive value of an offspring to its genetic parents increases with age, as the offspring approaches reproductive maturity. Both of these conditions would make an ancestral caretaker benefit, evolutionary speaking, from favoring older offspring over younger offspring when allocating their limited resources in parental investments. Third, if the prospects of fostering an offspring to reproductive maturity are not favorable, the caretaker might benefit from lowering or curtailing parental investment in the offspring as soon as possible. Limited resources may then be saved for parental investment in future offspring, assuming that the individual’s prospects might change and become more favorable toward successfully raising offspring.

From this line of adaptive logic, proponents of EP perspectives on filicide have predicted that there will be an increased risk for victimization among the youngest children (Daly and Wilson, 1988a,b, 1994; Buss, 2005; Duntley and Buss, 2011). There is empirical support for this EP prediction cross-culturally, as the highest rates for filicide victimization by caretakers are found to be during the first year of life (infanticide), and in particular during the first 24 h after the birth of the child (neonaticide), in societies such as the United States (Daly and Wilson, 1988a,b; Overpeck et al., 1998; Weekes-Shackelford and Shackelford, 2004; Shackelford et al., 2005, 2008), Canada (Daly and Wilson, 1988a,b; Bourget and Gagné, 2005; Harris et al., 2007), Australia (Alder and Polk, 2001; Nielssen et al., 2009), Fiji (Adinkrah, 2000, 2001, 2003), the United Kingdom (d’Orban, 1979; Marks and Kumar, 1993, 1996; Cavanagh et al., 2007; Flynn et al., 2007), Italy (Camperio Ciani and Fontanesi, 2012), Finland (Kauppi et al., 2010), and Sweden (Somander and Rammer, 1991; Temrin et al., 2004, 2011; Nordlund and Temrin, 2007).

Because only women gestate and nurse, thus investing high levels of their physiological resources directly in the youngest children, this adds a sex-differentiated selection pressure to the already significant selection pressure toward conditionally discriminating against the youngest offspring (Trivers, 1972, 1974). From this line of adaptive logic, proponents of EP perspectives on filicide have predicted that the youngest children will be at a greater risk for victimization by their mothers than by their fathers (Daly and Wilson, 1988a,b; Buss, 2005; Duntley and Buss, 2011). There is empirical support for this EP prediction cross-culturally. For instance, in a national sample of filicides in Finland, 1970–1994, mothers perpetrated 90% of the incidents where the victim was 1 year old or younger (Kauppi et al., 2010). Similar findings have been confirmed in other samples from Finland (Lehti et al., 2012), and in samples from the United States (Overpeck et al., 1998), Canada (Daly and Wilson, 1988b; Harris et al., 2007), Fiji (Adinkrah, 2000, 2001, 2003), the United Kingdom (d’Orban, 1979; Marks and Kumar, 1993, 1996; Flynn et al., 2007, 2013), and Sweden (Somander and Rammer, 1991; Nordlund and Temrin, 2007).

The increased risk of filicide victimization among the youngest children, and their particular risk at the hands of their mothers, was also confirmed in a national sample of filicides in Norway from the time period 1950–1979 (Grûnfeldt and Steen, 1984). Since that time period, Norwegian women’s options for safe, legal, and accessible family planning have increased dramatically. For instance, in a national survey on the use of birth control among sexually active women aged 20–44, who were not planning on becoming pregnant, 90% reported using at least one form of birth control in the past 3 months (Skjelstad, 2007). Further, Norway legalized abortion in 1978 and secured all women access to this medical procedure within the first 12 weeks of pregnancy through the publicly-funded national health care system. The findings from a register study of women living in Oslo, the capital city of Norway, who underwent an abortion in 2000–2002, reflect how these women used abortion for family planning with regard to their ability to provide for their young, as the women appeared to be terminating their pregnancies to suit their current and future circumstances with regard to completing their education and their employment status and to limit the number/frequency of children they birthed (Eskild et al., 2007).

If the EP hypothesis that filicide perpetration is underpinned by psychological mechanisms enabling discriminant parental investment is valid, there should be a notable drop in the frequency of victimization among the youngest children, and in particular among those victimized by their genetic mothers, associated with the increased ability for avoiding unwanted pregnancies and births. And this is the case in Norway. During the time period 1950–1979, there were 36 neonaticide cases, exclusively perpetrated by the victims’ mothers, identified in Norway (Grûnfeldt and Steen, 1984), but no officially confirmed incidents of neonaticide were identified in Norway during the time period 1990–2009 (Ottesen, 2012). Although there may have been unidentified incidents of neonaticide in the latter time period, and even identified incidents that were not registered and investigated due to prosecutorial discretion, the complete absence of officially confirmed neonaticide incidents suggests that the potential factual rate will currently be at a historic low.

The apparent absence of neonaticides in modern-day Norway coincides well with the significant decrease in filicide rates where the victims are 4 years old or younger that is recorded in other westernized countries, such as the other Nordic countries, the EU, the United States, Australia, and Japan since the mid-20th century (Resnick, 1970; Somander and Rammer, 1991; Janson et al., 2007; Lehti et al., 2012; Ottesen, 2012; Sturup and Granath, 2015). The decrease has been partly attributed to women’s increasing access to effective birth control and legal and safe abortion in these societies, and thus their ability to prevent unwanted pregnancies and births. These are proximate explanations of the decrease. One can add an ultimate level of explanation (i.e., evolutionary informed) to the current scholarly understanding of the decrease that effective, safe, accessible, and legal measures for family planning alleviate women from birthing children in conditions that are ancestrally salient cues for (over)activating psychological mechanisms for extreme discriminate parental investment.

### 2.2. Household compositions

As genes are the unit for evolutionary selection, it is theoretically argued and empirically confirmed within the field of evolutionary biology that selection processes will inevitably shape social behavior, including caretaker behaviors, to be nepotistic (i.e., favoring genetic relatives over others; Hamilton, 1964; Trivers, 1972; Alexander, 1979). Although it can be a successful mating strategy for the individual to make parental investments in an offspring their partner has from a previous union (e.g., Trivers, 1972), evolutionary selection processes will first and foremost favor psychological mechanisms that motivate individuals to discriminate between genetic and non-genetic dependents by investing more willingly and heavily in the former. From this line of adaptive logic, proponents of EP perspectives on filicide have predicted an increased risk for victimization in step-parental households compared to households with two genetic parents (Daly and Wilson, 1988a,b, 1994, 2001; Buss, 2005; Duntley and Buss, 2008, 2011). There is empirical support for this prediction cross-culturally. For instance, Daly and Wilson (1988a,b) found that victims were 100 times more likely to have lived with a stepparent than with two genetic parents in a sample of filicides that occurred in the city of Detroit (United States) in the year 1972, and 70 times more likely to have lived with a stepparent than with two genetic parents in a Canadian national sample of filicides from the time period 1974–1983. Similar findings have been confirmed in other samples from the United States (Weekes-Shackelford and Shackelford, 2004) and Canada (Daly and Wilson, 1994; Wilson et al., 1995; Harris et al., 2007), and in samples from Australia (Alder and Polk, 2001), England and Wales (Daly and Wilson, 1994; Wilson et al., 1995; Cavanagh et al., 2007; Flynn et al., 2013; Nobes et al., 2019), and Sweden (Somander and Rammer, 1991; Daly and Wilson, 2001; Temrin et al., 2004, 2011; Nordlund and Temrin, 2007).

The psychological mechanisms that may function toward discriminating against genetically unrelated children, and thus also increase the risk for filicide perpetration in step-parental households, may be feeling resentment at making such investments and perceiving a stepchild as a burden (Daly and Wilson, 1988a,b, 1994; Buss, 2005; Duntley and Buss, 2008, 2011). Daly and Wilson (1988b, 1994) suggested that due to the activation of such psychological mechanisms, and all else being equal, stepparents will be more prone than genetic parents to subjecting the children in their care to physical abuse. As physical abuse may turn lethal, Daly and Wilson further predicted that stepparents will therefore also perpetrate fatal physical abuse more often than genetic parents. Assuming that genetic parents will be more prone to having altruistic motives for their filicides, Daly and Wilson also predicted that filicidal genetic parents will more often chose methods that may limit their victims’ suffering. There is cross-cultural empirical support for this string of associated predictions. For instance, in a study using a Canadian national sample of filicides from the time period 1974–1990, Daly and Wilson (1994) found that stepfathers were not more likely than genetic fathers to shoot their victims, but they were 120 times more likely to beat their victims to death. Similar findings have been confirmed in other Canadian samples (Daly and Wilson, 1988b; Wilson et al., 1995; Harris et al., 2007) and in samples from the United States (Weekes-Shackelford and Shackelford, 2004), and England and Wales (Wilson et al., 1995; Cavanagh et al., 2007; Flynn et al., 2013).

If the EP hypothesis that filicide is perpetrated in the context of extreme discriminant investment is valid, one may expect the level of risk for step-parental filicide in a given society to be contingent on the extent to which caretakers are requested to invest in stepchildren and the extent to which that investment may be perceived as a burden. Daly and Wilson (2001) argued that if a society is structured so that stepparents do not experience heavy pseudo-parental obligations, the overrepresentation of step-parental filicide perpetrators will not be as significant as that found in societies where such obligations are indeed heavy. In modern-day Norway, genetic parents, mothers and fathers alike, have a legal right and strong social expectation to share custody of their children and are legally obligated to contribute financially toward the care of their children in the event of a relationship dissolutions. These rights, expectations, and obligations remain despite either of the genetic parents’ marriage to a new partner. There are thus both social and legal structures that contribute toward alleviating stepparents (including potential stepparents) from salient cues to an (over)activation of psychological mechanisms for discriminant parental investment.

Additionally, caretakers are prohibited from engaging in the most extreme and violent behavioral manifestation of any potential (over)activation of psychological mechanisms underpinning discriminant parental investment in modern-day Norway, as *The Child and Parent Act* (chapter 5, §30), implemented in the early 1980s, criminalized all forms of corporal punishment and physical discipline of children. A similar law, implemented in Sweden in 1979, is claimed to have raised awareness of the negative consequences of corporal punishment and elicited a change in the public’s attitudes toward children’s rights, and aided the social service’s opportunity to intervene upon suspicion of physical abuse—thus contributing toward both preventing the occurrence of physical abuse toward children in the general population and making fatal abuse almost non-existent in this society today (Somander and Rammer, 1991; Durrant, 1999; Durrant and Janson, 2005). Finland implemented a similar law in the early 1980s, and it is claimed to have had the same effect as in Sweden (Lehti et al., 2012).

If the EP hypothesis concerning filicide perpetration is valid, and assuming that evolved psychological mechanisms underpinning discriminant parental investment are contingent on ancestrally salient cues for (over)activation as well as on behavioral options for their specific manifestation, one may predict that step-parental perpetrators will not be as highly present in a national sample of filicides from modern-day Norway as they have been documented to be in samples drawn from the general population in societies where there both are heavier obligations toward pseudo-parental investment and where all forms of corporal punishment and physical disciplining of children have not been criminalized.

The single-parent household is a second household composition argued from EP perspectives to increase the risk for filicide perpetration that may be less relevant in modern-day Norway compared to other types of societies. Considering the amount of parental investment needed to foster a young infant and child, single parenthood and a lack of support from allo-parents may have been crucial cues to the ancestral caretaker of having poor prospects for successfully fostering the infant or child, and thus to withholding and even curtailing parental investment (Trivers, 1972). Additionally, a child from a previous union could arguably pose an impediment to future relationships for the single parent, as new partners would be hesitant to make parental investments in a stepchild, as already described. From this line of adaptive logic, proponents of EP perspectives on filicide have predicted an increased risk for perpetration by single parents (Daly and Wilson, 1988b; Buss, 2005; Duntley and Buss, 2011). There is empirical support for this prediction cross-culturally. For instance, in a study of infanticide in England and Wales, using a national sample from the time period 1996–2001, less than half of the maternal perpetrators were either married or cohabiting with the victim’s father (Flynn et al., 2007). Similar findings have been confirmed in other samples from England (d’Orban, 1979), and in samples from the United States (Overpeck et al., 1998; Lewis and Bunce, 2003; Stone et al., 2005; Friedman et al., 2005a), Canada (Daly and Wilson, 1988b; Harris et al., 2007), Australia (Alder and Polk, 2001), Fiji (Adinkrah, 2000, 2001), Italy (Camperio Ciani and Fontanesi, 2012), and Sweden (Temrin et al., 2004).

As already argued, the opportunities women have for preventing unwanted pregnancies and births have partly contributed toward a decrease in filicide victimization among the youngest children in several countries since the mid-20th century. In addition, a decrease in the social stigma of single parenthood (a stigma that could lead to a lack of allo-parental support and even an ostracization of the single mother from her family and society) and the introduction of progressive welfare benefits to single parents that compensate for the absent partner have also been argued to contribute toward the decrease in filicide victimization of the youngest children at the hands of their mothers (e.g., Resnick, 1970; Daly and Wilson, 2001; Lehti et al., 2012; Ottesen, 2012). Again, these are proximate explanations for the decrease. And, again, one can add an ultimate level of explanation to the current scholarly understanding of the decrease, as the listed social and political structures alleviate the individual from condition that could otherwise elicit an (over)activation of evolved psychological mechanisms for ancestrally adaptive discriminate parental investment. The social and political structures that alleviate single parents in this manner are well developed and highly progressive in modern-day Norway. If the EP hypothesis on filicide perpetration is valid, the risk for filicide perpetration by single parents should be lower in this society than that found in societies that are less progressive in these manners.

To summarize, the general population of modern-day Norway are alleviated from contexts that ancestrally held the potential for reproductive conflicts in caretaker-child-dyads and therefore also from cues that may (over)activate extreme discriminant parental investment to a greater extent than the societies in which EP perspectives on filicide have been tested so far. Further, laws criminalizing all forms of corporal punishment and physical disciplining of children limit caretakers’ opportunities to manifest the psychological mechanisms motivating for discriminant parental investment through the means of fatal abuse. Young victims and step-parental and single-parent households may therefore not be as characteristic of filicides in modern-day Norway as it is for filicides in other types of societies. This does not imply, however, that modern-day Norway will be *immune* to all categories of filicide perpetration. There is one subcategory of filicide that may still be as prevalent in this society as in other societies, namely those associated with perpetrator psychopathology, and EP perspectives give a theoretical foundation for predicting what traits will characterize this distinct subcategory.

### 2.3. Perpetrator psychopathology

Although current EP perspectives argue that filicide perpetration is a behavioral manifestation—albeit extreme—of psychological mechanisms that were selected for due to their function toward enabling ancestrally adaptive discriminant parental investment, this does not imply a dismissal of that filicides may at times occur due to perpetrator psychopathology. Indeed, from an EP perspective one may expect the perpetrator to suffer some form of severe psychopathology if the filicide is perpetrated in the absence of ancestrally salient cues to a reproductive conflict with the victim(s). In the vacuum of reproductive conflicts in caretaker-child-dyads in the general population in modern-day Norway, created by social, legal, and political structures as described in the above sections, one may predict that the majority of filicide perpetrators in this society will be suffering from some form of severe psychopathology.

From an evolutionary informed perspective, psychopathology may be understood as mental states that compromise the individual’s ability to perceive or act on its reproductive interests (Daly and Wilson, 1988b; Wilson et al., 1995; Stone et al., 2005). From this perspective, one may derive the hypothesis that filicides associated with perpetrator psychopathology will be characterized by traits that contradict an ancestrally adaptive logic for discriminant parental investment (Daly and Wilson, 1988b, 2001; Wilson et al., 1995; Stone et al., 2005; Harris et al., 2007; Duntley and Buss, 2008; Shackelford et al., 2008). This hypothesis enables the derivation of a series of predictions concerning precisely how the characteristics of filicides associated with perpetrator psychopathology will systematically differ from filicides perpetrated without such an association. In the following, four such predictions are presented.

As already presented, step-relations hold a greater potential for reproductive conflicts than genetic relations. Although there are conditions that elicit reproductive conflicts in genetic relations, as already presented, the genetic relationship will be a protective factor in caretaker-child dyad that is missing in the step-relations. Because it will more often be a greater—certainly a more direct—compromise to the individual’s reproductive interests to victimize a genetic offspring than a stepchild, one may predict that filicides perpetrated by genetic parents will be more likely to be associated with some severe form of psychopathology than those perpetrated by stepparents (Shackelford et al., 2008).

As already presented, a child’s reproductive value to its genetic parent increases as it approaches reproductive maturity. The potential compromise of perpetrating filicide to the individual’s reproductive interests will therefore increase with the victim’s age. One may therefore predict that filicides associated with some severe form of perpetrator psychopathology will be characterized by having older victims than filicides without this association (Stone et al., 2005; Shackelford et al., 2008).

Because fecundity decreases with age, the individuals’ prospects for future reproduction are reduced with age. Proponents of current EP perspectives of filicide have therefore predicted that filicide perpetrators will be relatively young, as they may afford to postpone making parental investments if their current prospects of succeeding are not promising (e.g., Daly and Wilson, 1988b). The older the individual gets, the riskier it would be to postpone making such investment, as losing a given child may constitute a greater compromise to the individual’s lifetime reproductive success. The individual is therefore expected to become less discriminative with regard to making parental investment in genetically related children with age. From this line of adaptive logic, one may predict that filicides associated with some severe form of perpetrator psychopathology will be characterized by having older perpetrators than filicides without this association (Stone et al., 2005; Shackelford et al., 2008).

Following the ancestrally adaptive logic implicit in the EP hypothesis that filicide is perpetrated in the context of reproductive conflicts in caretaker-child dyads, one would expect that such incidents will be characterized by having only a single victim, singled out by the caretaker due to being perceived as an excessive burden by ancestral standards to a life time reductive success. As multiple victims will constitute a greater potential compromise to the individual’s reproductive interests than singling out one victim, one may predict that filicides associated with some severe form of perpetrator psychopathology will have multiple victims more often than filicides without such an association (Shackelford et al., 2008).

The present study will disaggregate a presently compiled national sample from modern-day Norway depending on the presence of perpetrator psychopathology to explore whether the majority of filicides indeed are associated with some form of severe perpetrator psychopathology and further whether the two subcategories of filicide systematically differ as predicted. The following list presents the five predictions as research hypotheses to be tested against the present study’s data.

*Hypothesis 1*: The majority of filicides in modern-day Norway are perpetrated in association with some form of severe psychopathology.

*Hypothesis 2*: Genetic parents are more likely to suffer some form of severe psychopathology in association with their filicide perpetration than are step-parents.

*Hypothesis 3*: Filicides associated with some form of severe perpetrator psychopathology are more likely to have older victims than filicides without such an association.

*Hypothesis 4*: Filicides associated with some form of severe perpetrator psychopathology are more likely to have older perpetrators than filicides without such an association.

*Hypothesis 5*: Filicides associated with some form of severe perpetrator psychopathology are more likely to have multiple victims than filicides without such an association.

## 3. Materials

The present study defined filicide as caretaker perpetrated child homicide where the victims were <18 years old. The definition of caretaker included genetic parents, stepparents (including any current and former intimate partners of the genetic parent, such as boyfriends and girlfriends, as they were in the position of potentially becoming a stepparent in the conventional sense), and adoptive and foster parents. Incidents of premeditated and intentional murder and fatal abuse were included in the definition of homicide. The present study defined a perpetrator as either identified and convicted as such through conclusive court proceedings, including individuals adjudicated Not Guilty by Reason of Insanity (NGRI), or identified as the perpetrator through police investigations in incidents of filicide-suicide (see Ottesen (2016) for a further discussion of the listed definitions and inclusion criteria).

The time period chosen for the present study was 1990–2009. The time period that is covered then starts just over a decade after abortion was legalized and made accessible to the general population through public funding and just under a decade after corporal punishment and physical discipling was criminalized. The assumption is that these two clear shifts in social, political, and legal structures may influence the characteristics traits of filicide, as already argued, and the present study’s aim is to explore the applicability of EP predictions in modern-day Norway—a society where the general population is arguably alleviated from circumstances that may (over)activate extreme parental discrimination and has limited opportunities to manifest lethal caretaker behaviors.

Norway does not have a national homicide index intended for research. However, the National Criminal Investigation Service (NCIS) holds an archive of every single identified homicide incident committed in Norway. The NCIS is obliged to share information with the general public upon a formal request.^1^ The Norwegian penal code distinguishes premeditated and intentional murder from fatal abuse, dealing with the latter in a separate section. Incidents of fatal abuse are therefore not included in the NCIS’ homicide index, due to their adherence to a legal understanding of what constitutes a homicide. However, The National Police Computing and Material Services (NPCMS) hold an archive of all crimes registered by the police in association with an official police investigation in Norway, including incidents of fatal abuse, and were able to identify these filicides for the present study’s sample. The NCIS and NPCMS provided the present study with complementary lists of filicide incidents. Based on these lists, the respective police districts where the filicides occurred were contacted in order to access court verdicts.

In Norway, court verdicts include a narrative of the circumstances leading up to and surrounding the alleged criminal event, as well as detailed socio-demographic information about the defendants and victims. The information listed in the verdicts has been tried before the courts, where both the prosecution and the defense have argued for their narratives of the alleged crimes. The verdicts are therefore a relatively reliable and rich data source for collecting information concerning the characteristics of filicides in Norway. In incidents of filicide-suicide, socio-demographic information regarding the perpetrators and victims and short narratives authored by local police investigators were supplied by the staff at NCIS directly from their archive.

The Council for Professional Confidentiality and Research under the Ministry of Justice and Public Security, the Director of Public Prosecutions, and the Norwegian Social Science Services all approved the present study.

## 4. Results

There were 39 confirmed incidents of filicide with as many perpetrators in Norway during the years 1990–2009. In total, 48 children and 11 partners or former partners of the perpetrators were killed in these incidents. All 11 familicides had a male perpetrator, and all of these perpetrators were the genetic fathers of their victims. In the whole sample, 11 perpetrators were the genetic mothers of the victims, and 24 perpetrators were the genetic fathers of the victims.^2^ Additionally, there were four step-parental perpetrators. There were no foster or adoptive parents in the sample.

There is a striking support for *Hypothesis 1* in the present study’s sample, as the majority of filicides (79.5%) were associated with some form of severe psychopathology. Twenty-two perpetrators, of which 18 were paternal perpetrators, committed suicide in conjunction with the filicide. Two perpetrators, a genetic mother and a genetic father, attempted suicide in conjunction with their respective filicides. As they survived their suicide attempts, there are court verdicts in their respective cases, and these verdicts list that the perpetrators had psychiatric histories that included being diagnosed as clinically depressed and receiving mental health care prior to their filicide perpetration. Seven perpetrators, of which five were maternal perpetrators, were adjudicated psychotic at the time of their offense, and thus NGRI. Five of these perpetrators had a documented history of severe mental health issues, including presenting psychotic delusions, and contact with mental health services. Data on the mental health history were not available to the present study for two of the perpetrators adjudicated NGRI: In the following, the filicides of these combined 31 perpetrators will be referred to as filicides associated with perpetrator psychopathology.

There is also a striking support for *Hypothesis 2* in the present study’ sample, as there were no stepparents among the perpetrators who committed or attempted suicide in conjunction with their filicide or who were adjudicated psychotic, whereas 89% (31 of 35) of the genetic parents perpetrated their filicide in association with one of these forms of psychopathology.

Stepparents were excluded from the following analyses testing *Hypotheses 3–5* concerning the systematic differences between filicides with and without an association with perpetrator psychopathology. As already presented, it is theoretically and empirically established from EP perspectives that filicides perpetrated by genetic parents and stepparents are two distinct subcategories. This disaggregation of filicides depending on the genetic relationship between perpetrator and victim is also adhered to in conventional filicide research that does not explicitly apply EP perspectives (e.g., Friedman et al., 2005b).

To test *Hypothesis 3*, that filicides associated with some form of severe perpetrator psychopathology are more likely to have older victims than filicides without such an association, I used a cut-off age to categorize victims as either younger or older. Following previous research that has tested EP predictions concerning the victims’ age, the chosen cut-off age was 4 years (Daly and Wilson, 1988a,b, 1994, 2001; Temrin et al., 2000, 2004, 2011; Weekes-Shackelford and Shackelford, 2004; Shackelford et al., 2005, 2008; Nordlund and Temrin, 2007). In 22 (71%) incidents associated with perpetrator psychopathology, one or more of the filicide victims were above the cut-off age. Of the four incidents perpetrated by a genetic parent without an association with psychopathology, only one filicide victim (25%) was above the cut-off age. This finding supports *Hypothesis 3*.

There is also support for *Hypothesis 4*, that filicides associated with some form of severe perpetrator psychopathology are more likely to have older perpetrators than filicides without such an association, in the present study’s sample. The age of perpetrators of filicides associated with psychopathology ranged from 26 years to 51 years of age, with a mean age of 37.5 years. The age of the genetic parents without psychopathology ranged from 23 to 44 years of age, with mean age of 33.5 years.

Finally, *Hypothesis 5*, claiming filicides associated with some form of severe perpetrator psychopathology are more likely to have multiple victims than filicides without such an association was supported in the present study’s sample, as there were multiple victims in 12 (39%) of the 31 incidents associated with perpetrator psychopathology and in none of the four incidents perpetrated by a genetic parent without an association with psychopathology.

## 5. Discussion

Proponents of current EP perspectives on filicide have hypothesized that reproductive conflicts in caretaker-child-dyads may elicit psychological mechanisms that underpin potentially lethal levels of parental investment. From this hypothesis, they have derived predictions concerning the characteristic traits of filicide. If the hypothesis is valid, then the characteristics of filicide should follow an ancestrally adaptive logic for discriminant parental investment. However, as with all evolved psychological mechanisms, those underpinning discriminant parental investment will be contingent on ancestrally salient cues for their activation and available behavioral options for their specific manifestations. All three premises have implications for how we approach testing the universal validity of EP perspective on filicide in a theoretically sound and qualified manner.

Rather than setting an exact replication of findings from previous studies as the benchmark for the universal applicability of EP perspectives on filicide, the present study first considered whether evolved psychological mechanisms underpinning discriminant parental investment would be (over)activated and manifested in filicide perpetration in modern-day Norway, and argued that this is a society where social, legal, and political structures create a vacuum of salient cues to reproductive conflicts in caretaker-child-dyads in the general population and limit caretakers’ opportunities for lethal manifestations of such potential conflicts.

As predicted from current EP perspectives on filicide, the present study identified an extremely low occurrence of filicides that were not associated with perpetrator psychopathology. Over a 20-year period, in a population of <5 million people, only eight such incidents were officially registered (of which half were perpetrated by stepparents). This finding, along with the previously published finding of an absence of neonaticides in same time period (Ottesen, 2012), arguably reflects the alleviation of ancestrally salient cues to extreme discriminant parental investment in the general population and a lack of caretaker-behavioral options that may turn lethal in modern-day Norway, indirectly lending support to the EP hypothesis on filicide perpetration.

Closely associated with the EP hypothesis that filicide perpetration results from the (over)activation of evolved psychological mechanisms in the context of ancestrally salient cues to reproductive conflicts in the caretaker-child dyad, is the hypothesis that if caretakers perpetrate filicides in the absence of such cues, their filicides are likely to be associated with some severe form of psychopathology that is interfering with their ability to perceive or act in accordance with their reproductive interests. Arguably, all filicides could be said to interfere with the perpetrators’ reproductive interests in modern-day societies—certainly in a highly progressive welfare state like modern-day Norway. However, EP perspectives do not regard psychological mechanisms for their current adaptive value and function but for their ancestrally adaptive value and function. There may often be a mismatch between modern-day and ancestral societies with regard to what psychological mechanisms and mental states may undermine the individuals’ lifetime reproductive success, without arguably entailing psychopathology from an EP perspective. Rather, an EP perspective on psychopathology is that it entails mental states that would have severely interfered with the individual’s ability to perceive or act in accordance with its reproductive interests in our species’ ancestral past.

Assuming that the aforementioned vacuum of reproductive conflicts in caretaker-child dyads and opportunities for lethal responses to such conflicts indeed is created in modern-day Norway, it was predicted that filicides in modern-day Norway will characteristically be associated with severe perpetrator psychopathology. This prediction, presented and tested as *Hypothesis 1,* was supported as perpetrators who either perpetrated their filicide during a psychotic episode or in conjunction with their (attempted) suicide constituted 79.5% (31 of 39) of the whole sample.

Although it is common to disaggregate filicides depending on perpetrator psychopathology in conventional filicide research, this research does not reference any theoretical grounds for making such a disaggregation nor for predicting that the characteristic traits of the two suggested subcategories will differ in any systematic way. This is likely due to the fact that EP perspectives are unique among current theoretical approaches to the scholarly understanding of filicide in that they offer a comprehensive theoretical framework for making such a disaggregation and predicting precisely how the two subcategories will systematically differ in their characteristic traits. Just as the EP hypothesis concerning filicides in the context of reproductive conflicts in the caretaker-child dyad caters a theoretical ground for predicting that the characteristic traits of filicides will follow an ancestrally adaptive logic for discriminant parental investment, the EP hypothesis that filicides associated with perpetrator psychopathology will contradict adaptive logic also caters a theoretical ground for predicting what the characteristic traits of this subcategory necessarily will be. The present study tested four such predictions, presenting them as *Hypotheses 2–5*. The full set of predictions were supported in the present study’s sample, thus lending support to the EP perspectives they were derived from.

Although the compiled national sample was small, it can be argued that the findings from this sample reflect real and systematic universal differences between the two suggested subcategories of filicides, and are not merely random or local effects, as they all coincide with findings from previous filicide research. The support for *Hypothesis 2*, in that there were no stepparents who suffered from psychopathology in the present study’s sample whereas 89% (31 of 35) of the perpetrators who were the victims’ genetic parents did, coincides with a tendency that has been documented cross-culturally in larger samples. For instance, in a national sample of filicide in Sweden, 1965–1999, none of the stepparents committed filicide-suicide, whereas 40% of the genetic parents did (Nordlund and Temrin, 2007). Similar findings have been confirmed in other Swedish samples (Somander and Rammer, 1991), and in samples from the United States (Stone et al., 2005; Shackelford et al., 2008), Canada (Daly and Wilson, 1988b; Wilson et al., 1995; Bourget and Gagné, 2005; Harris et al., 2007), and England and Wales (Daly and Wilson, 1994; Wilson et al., 1995; Flynn et al., 2013). Deviating from this otherwise seemingly universally systematic difference between filicides perpetrated by genetic parents and stepparents, a study of filicides that occurred in Chicago (United States), 1965–1994, did not find a significant difference between the percentage of step-parental perpetrators who committed suicide (6.3%; 4 of 63) and genetic parents who also did so (6.1%; 24 of 396; Shackelford et al., 2005). There were, however, notably fewer suicides among genetic parents in their sample than that found in other studies, which arguably may be considered an equally remarkable anomaly.

When analyzing only filicides perpetrated by a genetic parent, the filicides associated with perpetrator psychopathology had older victims (defined as >4 years old) more often than filicides without such an association, which supports *Hypothesis 3*. This difference between the two suggested subcategories of filicides has also been documented cross-culturally in larger samples. For instance, in a national sample of filicides in Sweden, 1971–1980, the average age of victims of filicide-suicide was 6.5 years whereas the average age of victims of filicide without perpetrator suicide was 3.5 years (Somander and Rammer, 1991). Similar findings have been confirmed in samples from the United States (Shackelford et al., 2005, 2008; Stone et al., 2005; Friedman et al., 2005a,b), Canada (Daly and Wilson, 1988b; Bourget and Gagné, 2005; Harris et al., 2007), Australia (Alder and Polk, 2001), Italy (Camperio Ciani and Fontanesi, 2012), the United Kingdom (d’Orban, 1979; Marks and Kumar, 1993; Marks and Kumar, 1996; Flynn et al., 2007, 2013), and Finland (Kauppi et al., 2010).

When analyzing only filicides perpetrated by a genetic parent, filicides associated with perpetrator psychopathology had older perpetrators than filicides without such an association, which supports *Hypothesis 4*. This difference between the two suggested subcategories of filicides has also been documented cross-culturally in larger samples. For instance, in a study of filicides in Canada, 1974–1983, Daly and Wilson (1980) found that maternal perpetrators who committed filicide-suicide had a mean age of 29.5, whereas maternal perpetrators who did not commit suicide had a mean age of 22.5. Paternal perpetrators who committed filicide-suicide had a mean age of 30.5, whereas paternal perpetrators who did not commit suicide had a mean age of 25.8. Similar findings have been confirmed in another sample from Canada, 1996–2003 (Harris et al., 2007), the United States (Lewis and Bunce, 2003; Shackelford et al., 2005, 2008; Stone et al., 2005; Friedman et al., 2005a,b), Australia (Alder and Polk, 2001; Nielssen et al., 2009), Italy (Camperio Ciani and Fontanesi, 2012), England and Wales (d’Orban, 1979; Flynn et al., 2007, 2013), and Finland (Kauppi et al., 2010; Lehti et al., 2012).

Finally, when analyzing only the filicides perpetrated by a genetic parent, filicides associated with perpetrator psychopathology had more often multiple victims than filicides without such association, which supports *Hypothesis 5*. Again, this tendency has been documented cross-culturally in larger samples. For instance, in a filicide-suicide sample from the Cuyahoga County (United States), 1958–2002, 85% of the paternal perpetrators killed or attempted to kill all the children in their care, and 55% committed or attempted familicide (Friedman et al., 2005b). Similar findings have been confirmed in other samples from the United States (Lewis and Bunce, 2003; Shackelford et al., 2005, 2008; Friedman et al., 2005a), Canada (Daly and Wilson, 1988b; Wilson et al., 1995; Bourget and Gagné, 2005), the England and Wales (d’Orban, 1979; Wilson et al., 1995; Flynn et al., 2013), and Sweden (Somander and Rammer, 1991; Nordlund and Temrin, 2007).

Although it should be noted that there is no definitive association between suicide perpetration and conventional understandings of psychopathology, as an individual may be suicidal without having a history of presenting symptoms of mental illness, such as depression, the EP understanding of psychopathology as mental states interfering with the individual’s ability to perceive or act on its reproductive interests implies that suicidal ideation may be considered a form of psychopathology. It is then an empirical question whether the characteristic traits of filicides perpetrated by suicidal caretakers indeed do contradict, or follow, an ancestrally adaptive logic for discriminant parental investment. Not only did the filicides perpetrated by suicidal caretakers in the present study’s sample contradict an ancestrally adaptive logic for discriminant parental investment, the predicted characteristics for filicides associated with perpetrator psychopathology appeared more pronounced for filicides that occurred in conjunction with the perpetrator’s (attempted) suicide than those perpetrated during a psychotic episode. There is currently no theoretical framework, from EP perspectives or any other perspectives, suggesting either a gradient or categorical difference in the characteristics of filicide-suicide and filicides associated with other forms for perpetrator psychopathology, such as psychotic episodes. Whether the apparent stronger tendency for the characteristics of filicide-suicides to contradict adaptive logic than filicides associated with the perpetrators’ psychotic episode are a random or local result or part of a universally systematic pattern should be investigated further, both theoretically and empirically, in future studies.

One may want to consider whether the seven perpetrators who were adjudicated NGRI due to perpetrating their filicide during a psychotic episode were adjudicated as such because of the characteristics of their filicide (rather than that psychotic episodes may entail mental states that increase the vulnerability for manifesting behaviors that contradict ancestrally adaptive logic). However, to be adjudicated NGRI, the suspect has to undergo extensive observation and evaluation by at least two independent forensic psychiatrists/psychologists, and their report is reviewed by a national commission of experts. These forensic psychiatrists/psychologists have access to the suspect’s health records, and perform their evaluation as soon as possible upon the apprehension of the suspect. As mentioned in the results section, five of the perpetrators adjudicated NGRI and having perpetrated their filicides during psychotic episodes had a history of presenting psychotic delusions and receiving treatment from the mental health care services. It is also worth noting that in one of these five cases, the perpetrator was at first *not* adjudicated NGRI, only to be adjudicated so soon after upon further observations in prison, leading him to be transferred to compulsory psychiatric care. On the other hand, one of the two perpetrators adjudicated NGRI for whom the present study did not have access to data on their mental health history did not match any of the predictions concerning filicides associated with perpetrator psychopathology, as she was <30 years old and her sole victim was <2 years old.

It is a novel and evolutionary informed hypothesis that the present paper is presenting and testing, that filicides perpetrated in association with perpetrator psychopathology will be characterized by traits that contradict ancestrally adaptive logic. It is not a hypothesis that exists in the conventional scholarly literature on filicide, and that one may therefore expect the forensic psychiatrists and psychologists in Norway to be familiar with and to thus have followed systematically over the two decades that the present study’s sample covers. Further, as listed, the tendency for the characteristic traits of filicide to contradict adaptive logic appeared to be more typical for those incidents where the perpetrator was suicidal than where the perpetration occurred during a psychotic episode. As it all stands, it seems probable that the findings from the present study may reflect a real and systematic difference between filicides that is contingent on perpetrator psychopathology, as hypothesized from current EP perspectives on filicide.

## 6. Conclusion

Filicide is not an arbitrary phenomenon on a population level. There are distinct, cross-cultural patterns in the characteristic traits of filicides that can be identified when we disaggregate samples into theoretically meaningful subcategories. This suggests that filicide perpetration is underpinned by a universally shared parental psychology. If this parental psychology is indeed universally shared, it is most likely evolved, as is argued from current EP perspectives. The extent to which a given subcategory of filicide will present itself in a society may nevertheless vary. This variation is arguably also predictable from current EP perspectives, when adhering to the premises that the activation and likely manifestations of evolved psychological mechanisms will be contingent on social, legal, and political structures. The present study adds modern-day Norway to the variety of societies in which EP perspectives on filicide are applicable and thus validated. The theoretical and empirical work presented in this paper should therefore encourage the continued use of EP perspectives in filicide research, as there still are “white spots on the map” with regard to the range of societies where such perspectives have yet to be explicitly tested, or tested in a theoretically qualified manner.

Identifying theoretically meaningful subcategories of filicides, so as to identify their respective characteristic traits, is not mere intellectual folly. Our ability to prevent these fatal actions are dependent on such theoretical and empirical work. The social, legal, and political structures argued in this paper as contributing toward the prevention filicides in modern-day Norway were not implemented for this specific function. Other societies may however consider implementing or strengthening the listed structures with an explicit reference to an attempt to prevent filicides. Globally, as societies develop their social, legal, and political structures, for whatever reason, in directions that alleviate their general population from circumstances that are ancestrally salient cues for reproductive conflicts and limit the possibilities for lethal responses to such conflicts, an increasing proportion (i.e., not an increasing rate) of their filicides may be expected to be associated with perpetrator psychopathology. There is a relative invariance in the rate of homicides associated with perpetrator psychopathology across societies [from an EP perspective one could argue that this may be because homicides perpetrated in association with psychopathology is not contingent on ancestrally salient cues to reproductive conflict (i.e., not contingent on societal structures beyond those associated with psychiatric health care services)]. In societies where the homicide rate is low, the proportion of homicides associated with perpetrator psychopathology is therefore relatively increased (Coid, 1983; Daly and Wilson, 1988b. However, see Large et al. (2009) for a different perspective). This tendency has been found to extend to filicides (Daly and Wilson, 2001; Flynn et al., 2007, 2013; Lehti et al., 2012; Sturup and Granath, 2015). The success of the preventive measures for this specific subcategory of filicides will depend on scientifically acquired knowledge of its characteristic traits. The theoretical perspectives and empirical evidence presented in this paper suggest that filicides associated with perpetrator psychology will be characterized by a higher proportion of genetic relationships between perpetrator and victim, older perpetrators and victims, and having multiple victims more often than filicides without such an association.

## Data availability statement

The datasets presented in this article are not readily available because the dataset was required to be deleted upon end of analyses due to its sensitive nature. Requests to access the datasets should be directed to vibeke.ottesen@uib.no.

## Ethics statement

The studies involving human participants were reviewed and approved by the Council for Professional Confidentiality and Research under the Ministry of Justice and Public Security, the Director of Public Prosecutions, and the Norwegian Social Science Services. Written informed consent from the participants’ legal guardian/next of kin was not required to participate in this study in accordance with the national legislation and the institutional requirements.

## Author contributions

The author confirms being the sole contributor of this work and has approved it for publication.

## Conflict of interest

The author declares that the research was conducted in the absence of any commercial or financial relationships that could be construed as a potential conflict of interest.

## Publisher’s note

All claims expressed in this article are solely those of the authors and do not necessarily represent those of their affiliated organizations, or those of the publisher, the editors and the reviewers. Any product that may be evaluated in this article, or claim that may be made by its manufacturer, is not guaranteed or endorsed by the publisher.

## Footnotes

^1^An updated period beyond 2009 is not possible to include in the present sample and analyses as the National Police Directorate (NPD) has since 2013 instructed the National Criminal Investigation Service (NCIS) and The National Police Computing and Material Services (NPCMS) not to assist homicide research that is not politically initiated. After repeated requests for assistance in accessing data for research purposes from the homicide index were denied by the directorate, the present paper’s author lodged a formal complaint on the directorate’s instruction to the Ministry of Justice and Public Security in 2015, but the complaint was dismissed. A final request for the assistance from the NCIS was sent to the NPD in 2016, and was yet again declined.

^2^It should be noted that one genetic parent (not identified as suffering some form of severe psychopathology) believed he was being "cuckolded" into the paternal care of his victim – who he then subjected to fatal abuse. A DNA-test, taken in association with the police investigation to clarify this issue, revealed that he was, in fact, the genetic father of his victim.
